# Growth differentiation factor-15 as a potential biomarker in subclinical inflammation in familial mediterranean fever

**DOI:** 10.1007/s10067-026-08064-4

**Published:** 2026-03-30

**Authors:** Osman Cure, Merve Huner Yigit, Hakki Uzun, Huseyin Cinar Zihni, Ertugrul Yigit

**Affiliations:** 1 Department of Rheumatology, Faculty of Medicine, Recep Tayyip Erdogan University, 53000 Rize, Türkiye; 2 Department of Medical Biochemistry, Faculty of Medicine, Recep Tayyip Erdogan University, Rize, Türkiye; 3 Department of Urology, Faculty of Medicine, Recep Tayyip Erdogan University, Rize, Türkiye; 4 Department of Medical Biochemistry, Graduate School of Health Sciences, Karadeniz Technical University, Trabzon, Türkiye; 5 Department of Medical Biochemistry, Faculty of Medicine, Karadeniz Technical University, Trabzon, Türkiye

**Keywords:** Biomarker, Familial mediterranean fever, GDF-15, Subclinical inflammation

## Abstract

**Objectives:**

This study aimed to investigate whether growth differentiation factor-15 (GDF-15) can serve as a potential biomarker for assessing subclinical inflammation during the attack-free (intercritical) period in patients with familial Mediterranean fever (FMF).

**Methods:**

In a single-center cross-sectional case–control study, 52 FMF patients in the attack-free period were compared with 52 age- and sex-matched healthy controls. ELISA measured serum GDF-15 levels; acute-phase reactants (CRP, ESR, SAA, fibrinogen) and various hematologic inflammation indices were evaluated. Statistical analyses included the Mann–Whitney U, chi-square, Kruskal–Wallis, Spearman correlation, and ROC curve methods.

**Results:**

Serum GDF-15 levels were significantly higher in the FMF group than in controls (*p* < 0.001). Subclinical inflammation, defined by SAA > 10 mg/L, was detected in 78.8% of FMF patients. GDF-15 correlated positively with CRP and SAA (*p* < 0.05). GDF-15 levels did not differ across MEFV mutation subgroups or by the presence of the M694V mutation. Patients with subclinical inflammation had significantly higher GDF-15 levels than those without. ROC analysis showed that GDF-15 had a statistically significant ability to distinguish FMF from controls (AUC = 0.78; *p* < 0.001) and to identify subclinical inflammation (AUC = 0.74; *p* = 0.014).

**Conclusion:**

GDF-15 appears to be a potential biomarker reflecting ongoing subclinical inflammation during the attack-free period in FMF. Elevated GDF-15 levels in patients with SAA-defined subclinical inflammation suggest that GDF-15 may reflect low-grade inflammatory activity. Larger studies are needed to validate these findings.

**Table:** 

**Key Points** *• Serum GDF-15 was significantly higher in the attack-free FMF patients than in controls, supporting its potential to reflect persistent low-grade (subclinical) inflammation.* *• GDF-15 showed moderate discriminative performance (AUC 0.783) and may complement conventional acute-phase reactants in assessing inflammatory burden during attack-free periods.* *• GDF-15 levels did not differ significantly across MEFV mutation subgroups or by M694V status in this cohort.*

## Introduction

Familial Mediterranean Fever (FMF) is an autoinflammatory disease characterized by recurrent fever, serositis attacks, and arthritis [1, 2]. While the inflammatory response is markedly elevated during attacks, it has been shown that subclinical inflammation persists between attacks and increases the risk of complications such as amyloidosis [2, 3]. One study reported that subclinical inflammation in FMF is associated not only with amyloidosis development but also with chronic kidney disease, proteinuria, endothelial dysfunction, and increased cardiovascular risk; this condition was shown to correlate significantly with elevated erythrocyte sedimentation rate (ESR) detected in the attack-free periods and with the M694V mutation [4]. These complications affect both survival and quality of life in patients [2].

In addition to classical acute-phase reactants such as C-reactive protein (CRP), serum amyloid A (SAA), and fibrinogen, more accessible and low-cost hematologic indices—neutrophil-to-lymphocyte ratio (NLR), platelet-to-lymphocyte ratio (PLR), mean platelet volume (MPV), and red cell distribution width (RDW)—are also being investigated for assessing subclinical inflammation in FMF [3, 5]. In asymptomatic children with FMF, these parameters have been reported to be higher than in healthy controls, and MPV is associated with disease severity [3, 6, 7]. During attack-free periods in a cohort of 168 pediatric patients, elevated SAA (28.5%), CRP (13.6%), and ESR (20.8%) were detected, underscoring the limitations of classical markers [8]. Although laboratory findings are associated with subclinical inflammation, they do not correlate with MEFV mutations, indicating limited predictive value [9]. Moreover, normal CRP and SAA levels do not exclude the presence of inflammation; therefore, additional biomarkers are needed to complement classical markers.

Growth Differentiation Factor-15 (GDF-15) is a biomarker whose serum levels increase in states such as inflammation, cellular stress, tissue injury, and oxidative stress [10]. Elevated levels have been detected in chronic inflammatory conditions such as cardiovascular disease [11], chronic kidney failure [12], obesity [13], Alzheimer’s disease [14], inflammatory bowel diseases [15], and multiple sclerosis [16]. The finding that GDF-15 levels remain high even during clinically quiescent periods in patients with antiphospholipid syndrome and multiple sclerosis suggests that this biomarker has the potential to reflect subclinical inflammation [17, 18]. In addition, studies have reported a regulatory role for GDF-15 in inflammatory processes [18, 19]. To our knowledge, studies examining GDF-15 levels in FMF patients are limited. Therefore, this study evaluates serum GDF-15 as a potential biomarker of subclinical inflammation in FMF patients during the attack-free period.

## Materials and methods

### Study design and participants

This single-center, cross-sectional case–control study compared patients with FMF (*n* = 52) and healthy controls (*n* = 52). All FMF cases were sampled during the attack-free (intercritical) period; patients who had not experienced an acute attack within the preceding ≥ 2 weeks and who had no ongoing acute attack symptoms at the time of sampling were included. The interval since the last FMF attack was not captured in a standardized manner; therefore, the median time since the last attack could not be reported. Patients in the attack period were excluded primarily because GDF-15 may transiently rise during acute inflammatory responses, potentially affecting GDF-15 levels and complicating the assessment of subclinical inflammation. Given that the primary aim was to evaluate the clinically silent yet biologically active low-grade inflammatory state (subclinical inflammation) between attacks in FMF, measurements obtained during acute phases were considered unlikely to reflect this pathophysiology. Therefore, inclusion of only patients in the attack-free period was a methodological choice to more accurately assess the potential of GDF-15 as a biomarker of low-grade, chronic inflammation.

All FMF patients had been followed for at least one year after diagnosis, with a mean disease duration of 12.5 years. All were on regular colchicine therapy, with doses ranging from predominantly 1–1.5 mg/day. Healthy controls were volunteers matched to the patient group by age and sex. Based on a sensitivity analysis conducted with G*Power (v3.1), the total sample size of *n* = 104 (FMF = 52, control = 52) provided 80% power to detect medium between-group differences (Cohen’s d ≈ 0.55). For the comparison within the FMF cohort between those with (*n* = 41) and without (*n* = 11) subclinical inflammation, the minimum detectable effect size was estimated at d ≈ 0.95 (large effect), indicating that this subgroup analysis is sufficiently powered only for large differences.

### Ethical approval

The study protocol was approved by the Recep Tayyip Erdoğan University Clinical Research Ethics Committee (Approval No.: 2024/53; Reference: E-40465587–050.01.04–989; Approval Date: 14 March 2024). Written informed consent was obtained from all participants prior to enrollment.

### Inclusion and exclusion criteria

Inclusion Criteria: (i) Diagnosis of FMF according to the Livneh (Tel Hashomer) criteria, based on major and minor clinical findings [20]. (ii) Age 18–65 years. (iii) The attack-free period at the time of sampling (no acute attack within the preceding ≥ 2 weeks).

Exclusion Criteria: (i) Presence of a concomitant active or chronic infection, malignancy, advanced hepatic/renal failure, or diabetes mellitus; (ii) pregnancy; (iii) use of systemic corticosteroids and/or immunosuppressive biologic therapies that could affect biomarker levels (colchicine excluded) [21].

### Clinical data collection

Demographic data (age, sex), age at diagnosis and disease duration (time from diagnosis to sampling), smoking/alcohol use, body mass index (BMI), history of typical attack manifestations symptoms (fever, abdominal/chest pain, arthritis), and colchicine dose (as recorded in charts) were extracted from medical records using a standardized form. Disease activity was assessed using the Auto-Inflammatory Diseases Activity Index (AIDAI) and the International Severity Scoring System (ISSF) for FMF [22, 23].

### Biochemical and acute-phase reactant analyses

Biochemical tests were performed on an AU5800 automated analyzer (Beckman Coulter, Brea, CA, USA) in accordance with the manufacturer’s procedures and the laboratory’s routine internal quality controls. The panel included glucose, BUN, creatinine, eGFR, uric acid, lipid profile (TC, TG, HDL-C, LDL-C, VLDL), liver enzymes (ALP, ALT, AST, GGT, LDH), and minerals (calcium, magnesium, phosphate). Acute-phase reactants comprised CRP, ESR, SAA, and fibrinogen; immunoturbidimetric methods measured SAA and fibrinogen.

### Hematological parameter analyses

Complete blood counts were performed on a BC-6000 analyzer (Mindray, Shenzhen, China). Reported parameters included WBC, LY#, MO#, NE#, EO#, BA#; erythrocyte indices (RBC, HGB, HCT, MCV, MCH, MCHC, RDW-SD, RDW-CV); and platelet parameters (PLT, MPV, PCT, PDW).

### Serum GDF-15 measurement

Serum GDF-15 levels were measured in duplicate using the Quantikine® Human GDF-15 ELISA kit (R&D Systems; Cat. No.: DGD150). Absorbance was read at 450 nm (with a 540/570 nm reference), and concentrations were calculated from a recombinant standard curve using a four-parameter logistic (4-PL) fit.

### Derived inflammatory indices and hepatic fibrosis scores

To assess inflammatory burden, several ratios and indices derived from complete blood count parameters were used. NLR was calculated by dividing the neutrophil count by the lymphocyte count (NE#/LY#); MLR by dividing the monocyte count by the lymphocyte count (MO#/LY#); and PLR by dividing the platelet count by the lymphocyte count (PLT/LY#) [24]. More complex parameters included the systemic immune-inflammation index (SII), obtained by dividing the product of platelet and neutrophil counts by the lymphocyte count (PLT × NE#)/LY#; the systemic inflammation response index (SIRI), calculated by dividing the product of neutrophil and monocyte counts by the lymphocyte count (NE# × MO#)/LY#; and the aggregate index of systemic inflammation (AISI), calculated by dividing the product of neutrophil, monocyte, and platelet counts by the lymphocyte count (NE# × MO# × PLT)/LY# [25].

Additionally, three different scores were computed as indicators of hepatic fibrosis. The Fibrosis-4 (FIB-4) index was calculated as (Age × AST)/(PLT × √ALT); the Aspartate Aminotransferase-to-Platelet Ratio Index (APRI) as {[(AST/AST-ULN) × 100]/PLT}; and the aspartate aminotransferase/alanine aminotransferase ratio (AAR) as AST/ALT [26–28].

### Genetic subgrouping and definitions

MEFV genetic status was obtained from medical records and classified as no mutation/single heterozygous/homozygous/compound heterozygous; the presence of the M694V variant was additionally coded as absent/heterozygous/homozygous [29]. Subclinical inflammation was defined as SAA above the laboratory upper reference limit in the absence of attack symptoms (SAA > 10 mg/L) [3, 8]; as reported in the results, this variable was used in subgroup comparisons.

### Statistical analysis

All statistical analyses were performed using IBM SPSS Statistics for Windows, version 23 (IBM Corp., Armonk, NY, USA) and OriginPro 2025 (OriginLab Corp., Northampton, MA, USA) for data visualization. Continuous variables were tested for normality using the Shapiro–Wilk test. Because most variables did not meet the assumption of normality, nonparametric methods were used. Continuous data were summarized as median (minimum–maximum), median (Q1-Q3) or median (IQR), and categorical variables as frequency and percentage. For comparisons between FMF patients and controls, the Mann–Whitney U test was used for continuous variables, while categorical variables were analyzed with the Chi-square test. Comparisons across MEFV mutation subgroups and M694V genotypes were performed using the Kruskal–Wallis test, followed by Dunn’s post-hoc test for pairwise analyses. Correlations between circulating GDF-15 and clinical or laboratory parameters were evaluated using Spearman’s rank correlation coefficient (ρ). Significance was set at *p* < 0.05, and correlation heatmaps highlighted statistically significant associations. The discriminative/classification performance of biomarkers was assessed using the receiver operating characteristic (ROC) curve analysis. ROC curves were constructed to evaluate: (i) the ability of GDF-15 to discriminate FMF patients from healthy controls, and (ii) the diagnostic performance of CRP, SAA, fibrinogen, and GDF-15 for detecting subclinical inflammation (defined as SAA > 10 mg/L) within the FMF cohort. From each ROC analysis, the area under the curve (AUC), 95% confidence intervals (CI), and asymptotic *p*-values were reported. All tests were two-tailed, and a *p*-value < 0.05 was considered statistically significant.

## Results

### Demographic, clinical, and biochemical characteristics of study population

The demographic and clinical characteristics of the FMF (*n* = 52) and control (*n* = 52) groups are summarized in Table 1. No statistically significant differences were observed between the FMF and control groups in key demographic features such as age, sex, and body measurements, nor in many biochemical parameters. BMI was comparable between the FMF and control groups. In contrast, serum uric acid levels were markedly higher in the FMF group than in controls. Total leukocyte counts were also significantly higher in FMF patients; specifically, increases were noted in absolute lymphocyte, monocyte, and basophil counts. Red blood cell parameters also differed between groups: the mean erythrocyte count was higher in FMF patients, while the erythrocyte indices MCV and MCH were slightly lower, and RDW values were significantly higher. Additionally, HDL-C levels were significantly lower in the FMF group compared with the controls.

**Table 1:** Demographic and clinical characteristics of the study population

Parameters	Control (*n* = 52) Median (Min–Max)	FMF (*n* = 52) Median (Min–Max)	p *Chi-Square †Mann–Whitney U
Age (years)	32.5 (19–43)	35 (18–59)	†p > 0.05
Sex [female (*n*)/male (*n*)]	34/18	30/22	*p > 0.05
Smoking [yes (*n*)/no (*n*)]	14/38	11/41	*p > 0.05
Alcohol consumption [yes (*n*)/no (*n*)]	1/51	1/51	*p > 0.05
Weight (kg)	76 (50–110)	73.5 (48–110)	†p > 0.05
Height (cm)	165.5 (146–185)	166.5 (150–187)	†p > 0.05
BMI (kg/m^2^)	27.1 (18.3–38.5)	24.9 (17.7–37.6)	†p > 0.05
Glucose (mg/dL)	90 (75–113)	91.5 (70–178)	†p > 0.05
BUN (mg/dL)	24 (13–40)	26 (14–64)	†p > 0.05
Creatinine (mg/dL)	0.715 (0.26–1.09)	0.74 (0.43–1.89)	†p > 0.05
eGFR (mL/min/1.73 m^2^)	114 (81–132)	110 (31–149)	†p > 0.05
Uric acid (mg/dL)	4.5 (1.6–8)	4.9 (2.9–8.7)	†p = 0.029
Total protein (g/L)	75.65 (67.5–89.5)	76.6 (65.8–86.6)	†p > 0.05
Albumin (g/L)	46.8 (41.9–55.1)	46.4 (35.8–53.3)	†p > 0.05
TC (mg/dL)	205 (119–269)	193.5 (112–397)	†p > 0.05
TG (mg/dL)	98.4 (46–374)	118 (49–462)	†p > 0.05
HDL-C (mg/dL)	55.6 (27.6–90.1)	48.8 (27.3–85.2)	†p = 0.016
LDL-C (mg/dL)	130 (39–190)	121 (55–226)	†p > 0.05
Calcium (mg/dL)	10.1 (9.1–10.8)	10 (9.1–11.9)	†p > 0.05
Magnesium (mg/dL)	2 (1.7–2.2)	2.01 (1.64–2.31)	†p > 0.05
Phosphate (mg/dL)	3.4 (2.6–5.9)	3.6 (2.5–4.95)	†p > 0.05
WBC (× 10⁹/L)	6.5 (4.2–11)	7.3 (4.1–13.6)	†p = 0.02
LY# (× 10⁹/L)	1.96 (1.1–4.3)	2.28 (1.13–5)	†p = 0.034
MO# (× 10⁹/L)	0.37 (0.17–1.39)	0.41 (0.25–0.79)	†p = 0.021
NE# (× 10⁹/L)	3.735 (1.93–6.79)	4.6 (1.6–11.2)	†p > 0.05
EO# (× 10⁹/L)	0.14 (0–0.57)	0.15 (0.02–0.96)	†p > 0.05
BA# (× 10⁹/L)	0.03 (0.01–0.07)	0.03 (0.01–0.1)	†p = 0.009
RBC (× 10^12^/L)	4.63 (3.75–5.55)	4.83 (4.04–5.91)	†p = 0.039
HGB (g/dL)	13.6 (10.5–17.1)	14.05 (12–17)	†p > 0.05
HCT (%)	40.3 (32.2–49.1)	41.6 (36.1–49.7)	†p > 0.05
MCV (fL)	87.8 (76.6–96.9)	85.2 (78.6–95.8)	†p = 0.031
MCH (pg)	29.6 (25–33.5)	28.6 (24.8–32.1)	†p = 0.045
MCHC (g/dL)	33.6 (31.7–35.8)	33.6 (31.4–35.9)	†p > 0.05
PLT (× 10⁹/L)	273 (162–431)	277 (163–498)	†p > 0.05
MPV (fL)	10.5 (8.1–15.5)	10.3 (8.5–13.3)	†p > 0.05
PCT (%)	0.27 (0.19–0.42)	0.28 (0.19–0.48)	†p > 0.05
PDW (%)	16 (15.6–17.2)	16.1 (15.5–16.6)	†p > 0.05
RDW-SD (fL)	44.3 (41–52.7)	45.6 (37–57.4)	†p = 0.015
RDW-CV (%)	13.5 (12.2–16.2)	13.7 (12.2–16.9)	†p = 0.003

*BMI* body mass index, *BUN* blood urea nitrogen, *eGFR* estimated glomerular filtration rate, *TC* total cholesterol, *TG* triglycerides, *HDL-C* high-density lipoprotein cholesterol, *LDL-C* low-density lipoprotein cholesterol, *LY#* absolute lymphocyte count, *MO#* monocyte count, *NE#* neutrophil count, *EO#* eosinophil count, *BA#* basophil count, *RBC* red blood cells, *HGB* haemoglobin, *HCT* haematocrit, *MCV* mean corpuscular volume, *MCH* mean corpuscular haemoglobin, *MCHC* mean corpuscular hemoglobin concentration, *PLT* platelets, *MPV* mean platelet volume, *PCT* plateletcrit, *PDW* platelet distribution width, *RDW-SD* red cell distribution width–standard deviation, *RDW-CV* red cell distribution width–coefficient of variation

### Clinical features and disease characteristics of FMF patients

The clinical features and disease characteristics of FMF patients are presented in Table 2. The median disease duration exceeded ten years, and the median age at diagnosis was in the mid-twenties. Genetic analysis showed that the vast majority of cases carried at least one MEFV gene mutation. The most common genotype was a single heterozygous mutation, present in more than half of the patients. A homozygous mutation was identified in approximately one quarter, while compound heterozygosity was detected in fewer than 10%. A small subgroup (~ 10%) had no identifiable MEFV mutation. The leading clinical manifestations during attacks were abdominal pain (in about half of the cases) and fever (in more than one third), while chest pain and arthritis were reported less frequently. Median disease severity scores were 4 for ISSF and 5 for AIDAI. Serious complications were rare in the study cohort; only one patient had amyloidosis. By contrast, evidence of subclinical inflammation was present in roughly 80% of patients. All patients were on regular colchicine therapy; approximately two-thirds used 0.5 mg twice daily, and the remaining one-third used 0.5 mg three times daily.

**Table 2:** Clinical features and disease characteristics of FMF patients

Clinical Features	FMF Patients (*n* = 52)
Disease Duration (years), median (IQR)	11.5 (13.5)
Age at Diagnosis (years), median (IQR)	24.5 (13)
MEFV Mutations, n (%)	
No mutation	5 (9.6%)
Single heterozygous mutation	30 (57.7%)
Homozygous mutation	13 (25%)
Compound heterozygous mutation	4 (7.7%)
M694V mutation, n (%)	
Absent	32 (61.5%)
Heterozygous	8 (15.4%)
Homozygous	12 (23%)
Typical attack manifestations (history), n (%)	
Fever	18 (34.6%)
Abdominal pain	24 (46.1%)
Chest pain	12 (23%)
Arthritis	10 (19.2%)
Disease Severity	
ISSF score, median (IQR)	4 (1.25)
AIDAI score, median (IQR)	5 (2)
Complications, n (%)	
Amyloidosis	1 (1.9%)
Subclinical inflammation	41 (78.8%)
Treatment	
Colchicine dose (2 × 1)	34 (65.2%)
Colchicine dose (3 × 1)	18 (34.8%)

*ISSF* International Severity Scoring System for Familial Mediterranean Fever, *AIDAI* Autoinflammatory Diseases Activity Index, *MEFV* Mediterranean Fever gene, *M694V* mutation in the MEFV gene

### Laboratory parameters: comparison between groups

Biochemical liver enzymes and inflammatory parameters were compared between FMF patients and controls (Table 3). Baseline liver enzyme levels were notably higher in the FMF group than in controls. Specifically, ALP, ALT, AST, and GGT activities were significantly increased in FMF, whereas LDH levels were similar between groups. Among acute-phase reactants, CRP, fibrinogen, and SAA were significantly higher in FMF patients compared with controls. No statistically significant difference was observed for ESR (*p* > 0.05). Additionally, inflammatory indices such as NLR, MLR, PLR, SII, SIRI, and AISI were comparable between the FMF and control groups, with no significant differences detected.

**Table 3:** Enzyme markers and acute phase reactants of FMF patients and controls

Parameters	Control (*n* = 52) Median (Min–Max)	FMF (*n* = 52) Median (Min–Max)	*p* Mann–Whitney U
Enzymes Markers			
ALP (U/L)	64 (16–100)	82 (32–207)	***p* = 0.001**
ALT (U/L)	14 (7–72)	23 (7–254)	***p* = 0.006**
AST (U/L)	17 (11–33)	20 (11–157)	***p* = 0.009**
GGT (U/L)	17 (9–72)	23.5 (10–255)	***p* = 0.002**
LDH (U/L)	172 (127–217)	179 (114–304)	*p* > 0.05
Acute Phase Reactants			
CRP (mg/dL)	1.32 (0.2–12.73)	9.25 (0.2–93)	***p* = 0.001**
ESR (mm/h)	6.73 (2–22)	10.5 (2–39)	*p* > 0.05
Fibrinogen (mg/dL)	283 (175–403)	309 (208–729)	***p* = 0.002**
SAA (mg/L)	9.9 (2–25.8)	17.5 (2–185)	***p* = 0.001**
Inflammatory Indices			
NLR	1.8 (0.24–5.5)	2 (0.6–6.2)	*p* > 0.05
MLR	0.18 (0.02–0.7)	0.18 (0.01–0.52)	*p* > 0.05
PLR	131.7 (54.6–266.6)	121 (68–239.8)	*p* > 0.05
SII	534.7 (124.8–1826)	548 (187–1644)	*p* > 0.05
SIRI	0.64 (0.19–2.6)	0.9 (0.18–3.01)	*p* > 0.05
AISI	191.9 (58.2–627.44)	226.6 (48–816.4)	*p* > 0.05
Hepatic Fibrosis Score			
FIB-4	0.5 (0.23–1.36)	0.5 (0.24–1.78)	*p* > 0.05
APRI	0.12 (0.07–0.36)	0.15 (0.06–1.33)	***p* = 0.04**
AAR	1.09 (0.43–2.42)	0.91 (0.35–2)	*p* > 0.05

*ALP* alkaline phosphatase, *ALT* alanine aminotransferase, *AST* aspartate aminotransferase, *GGT* γ-glutamyl transferase, *LDH* lactate dehydrogenase, *CRP* C-reactive protein, *WBC* white blood cell count, *ESR* erythrocyte sedimentation rate, *SAA* serum amyloid A, *NLR* Neutrophil-to-Lymphocyte Ratio, *MLR* Monocyte-to-Lymphocyte Ratio, *PLR* Platelet-to-Lymphocyte Ratio, *SII* Systemic Immune-Inflammation Index, *SIRI* Systemic Inflammation Response Index, *AISI* Aggregate Index of Systemic Inflammation; Hepatic Fibrosis Score, composite score evaluating liver fibrosis, *FIB-4* Fibrosis-4 Index, *APRI* Aspartate Aminotransferase-to-Platelet Ratio Index, *AAR* Aspartate Aminotransferase-to-Alanine Aminotransferase Ratio

### Circulating GDF-15 levels in FMF vs controls

**Fig. 1:**
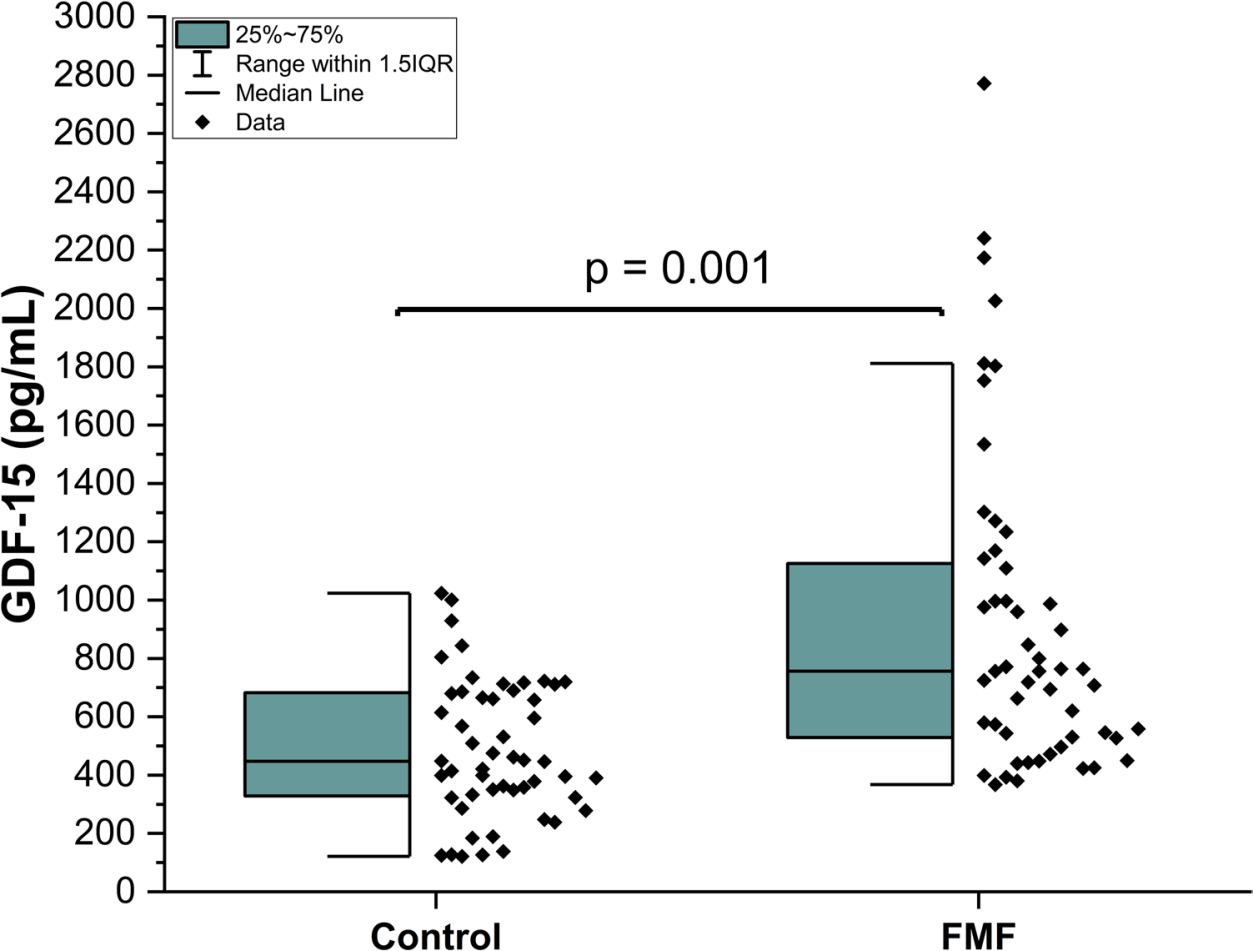
Circulating GDF-15 levels in FMF patients and healthy controls (Mann–Whitney U test)

The median circulating GDF-15 concentration was significantly higher in the FMF group than in healthy controls (Fig. 1).

### GDF-15, SAA, and fibrinogen by MEFV genotype, M694V status, and subclinical inflammation

Within the FMF cohort, GDF-15, SAA, and fibrinogen levels across genetic subgroups are presented in Table 4. In addition, GDF-15, SAA, and fibrinogen values stratified by subclinical inflammation status are shown in Table 5. No statistically significant differences were detected in GDF-15, SAA, or fibrinogen levels across MEFV genotype subgroups. Similarly, the absence, heterozygous presence, or homozygous presence of the M694V mutation did not significantly affect these biomarkers. By contrast, FMF patients with evidence of subclinical inflammation had significantly higher GDF-15 and SAA levels than those without subclinical inflammation, whereas fibrinogen values did not differ significantly between the two groups.

**Table 4:** GDF-15, SAA, and fibrinogen by MEFV genotype and M694V status in FMF (median [Q1–Q3])

	*n*	GDF-15 [Median (Q1-Q3)]	SAA [Median (Q1-Q3)]	Fibrinogen [Median (Q1-Q3)]
MEFV mutation status				
No mutation	5	450 (414.3–1098.8)	32 (3.44–42.2)	312 (280–502.5)
Single heterozygous mutation	30	805.7 (554.5–1186.0)	16.6 (11.1–26.3)	318.5 (279.2–387.7)
Homozygous mutation	13	756.5 (521.1–991.9)	18 (12.2–24)	293 (234.5–339.3)
Compound heterozygous mutation	4	618.1 (437.2–1549.9)	18.9 (12.2–43)	283.5 (261.25–452)
M694V mutation				
Absent	32	737.6 (504.0–996.5)	17.1 (9.87–25.75)	325.5 (248.7–377)
Heterozygous	8	985 (658.6–2068.7)	17.15 (5.77–24.2)	304 (280.2–373.5)
Homozygous	12	693.9 (490.5–1362.6)	20 (12.5–39.5)	299 (280.7–321.7)

*FMF* familial Mediterranean fever, *MEFV* Mediterranean fever gene, *M694V* Met694Val variant, *GDF-15* growth differentiation factor-15 (pg/mL), *SAA* serum amyloid A (mg/L). Data are presented as median (Q1–Q3). For inference, use the Kruskal–Wallis test with Dunn’s multiple comparisons of MEFV and M694V

**Table 5:** GDF-15, SAA, and fibrinogen by subclinical inflammation in FMF (median [Q1–Q3])

	*n*	GDF-15 [Median (Q1-Q3)]	SAA [Median (Q1-Q3)]	Fibrinogen [Median (Q1-Q3)]
Subclinical inflammation				
Yes	41	847.1 (544.7–1253)*	21 (15.9–30.4)*	310 (278.5–371)
No	11	558.1 (450–708)	4.72 (2.89–8.39)	290 (247–385)

The Mann–Whitney U test (*statistically significantly higher)

### Correlation analysis of GDF-15

**Fig. 2:**
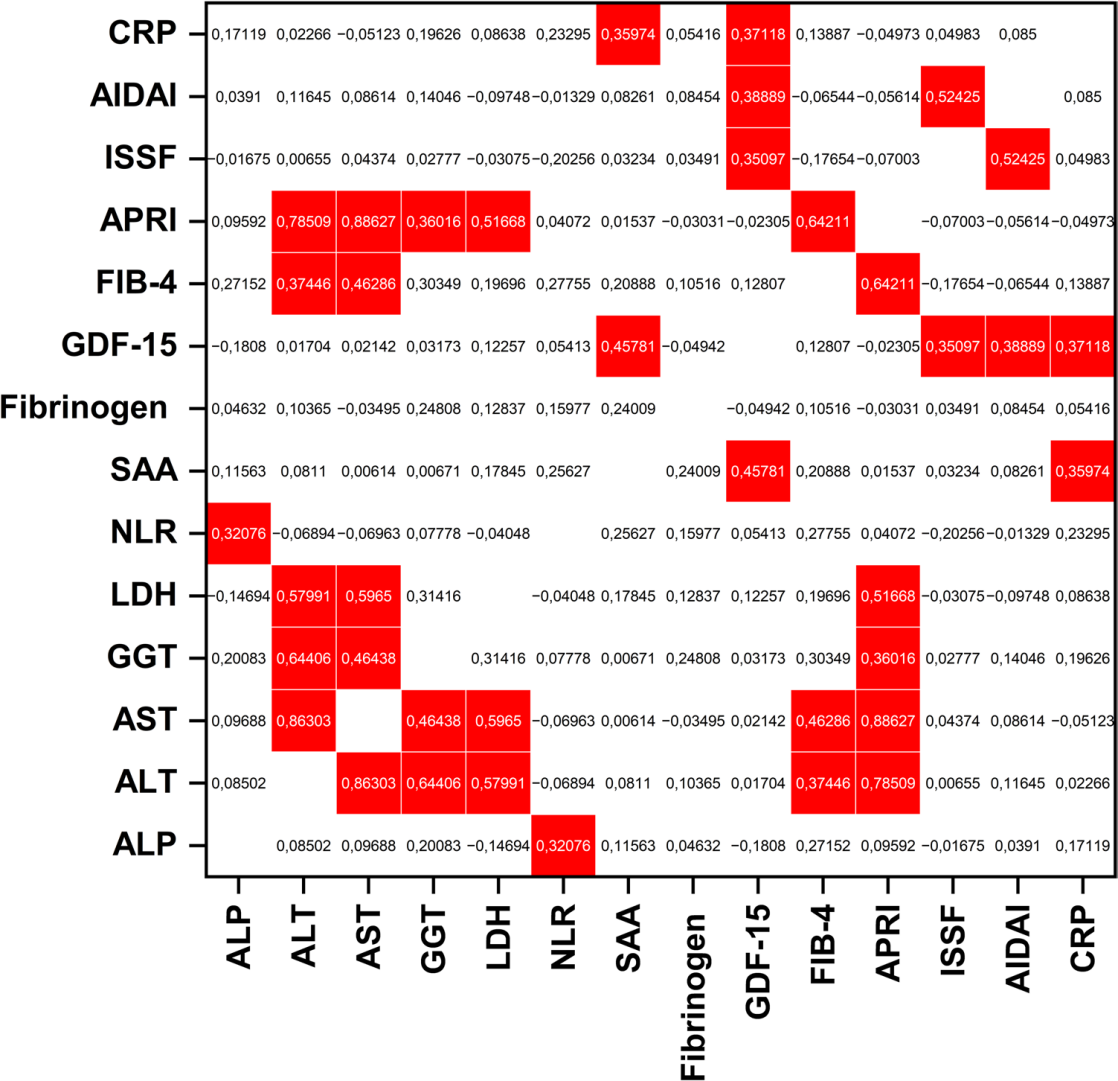
Spearman correlation matrix depicting associations between GDF-15 and various clinical/laboratory variables in FMF patients; cells in red indicate statistically significant correlations (*p* < 0.05)

In FMF patients, Spearman correlation analysis revealed significant associations between GDF-15 levels and classical inflammatory markers. In particular, GDF-15 showed positive and significant correlations with CRP and SAA (*p* < 0.05). By contrast, no notable relationships were observed between GDF-15 and liver enzymes (e.g., ALT, AST) or other laboratory parameters such as NLR. However, there was statistically significant correlation between GDF-15 levels and FMF disease severity scores (ISSF and AIDAI) (Fig. 2).

### Discriminative performance of GDF-15 in distinguishing FMF and controls

**Fig. 3:**
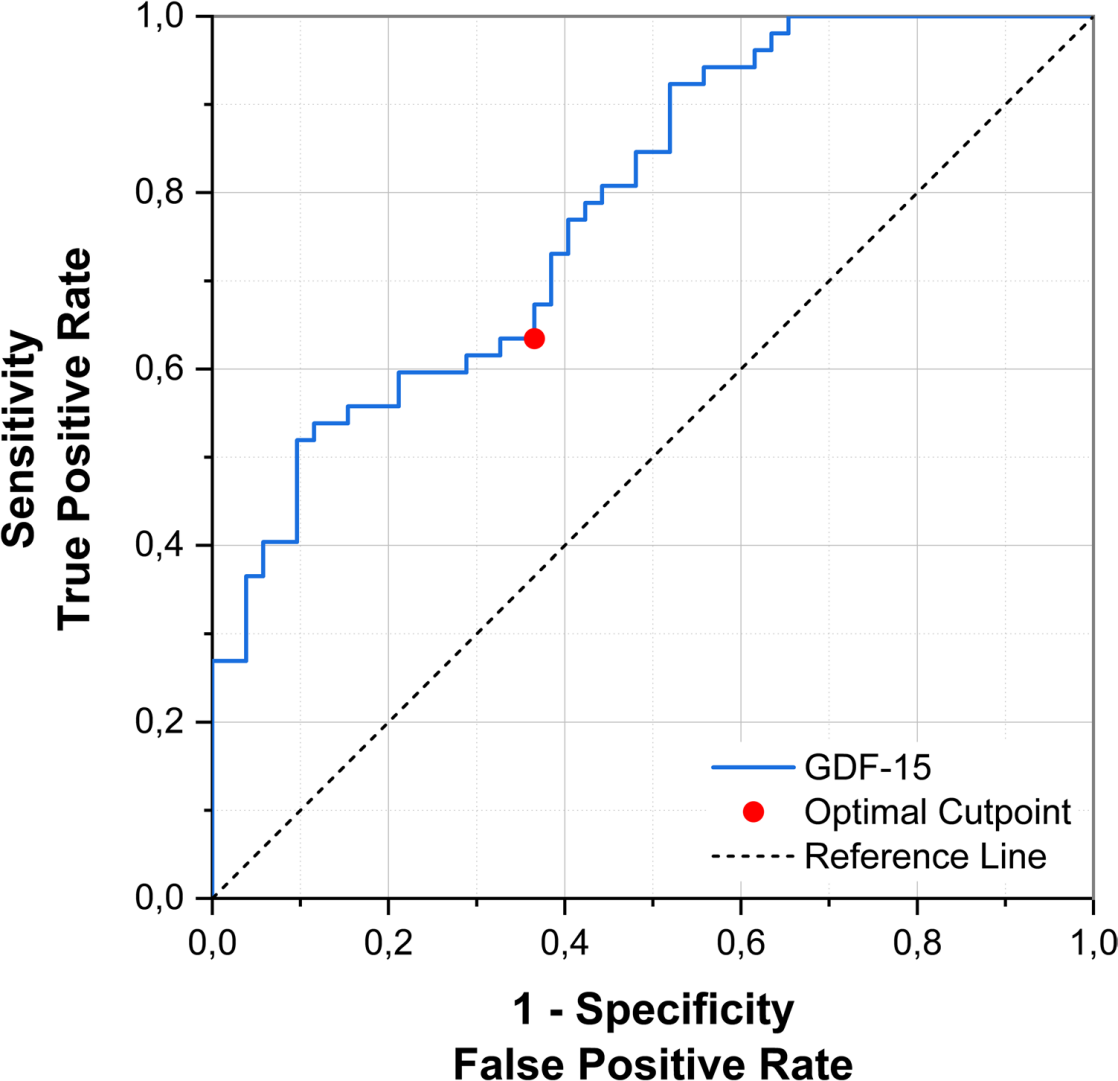
Receiver operating characteristic (ROC) curve for GDF-15 distinguishing FMF cases (*n* = 52) from healthy controls (*n* = 52). The curve has an area under the curve of 0.783 (95% CI 0.698–0.868; *p* < 0.001)

ROC analysis demonstrated that circulating GDF-15 levels possess a statistically significant ability to distinguish FMF patients from healthy controls. The area under the ROC curve (AUC) for GDF-15 was 0.783 (95% CI: 0.698–0.868), indicating significant discriminative power (*p* < 0.001) (Fig. 3).

### Discriminative performance of biomarkers for identifying subclinical inflammation in FMF

**Fig. 4:**
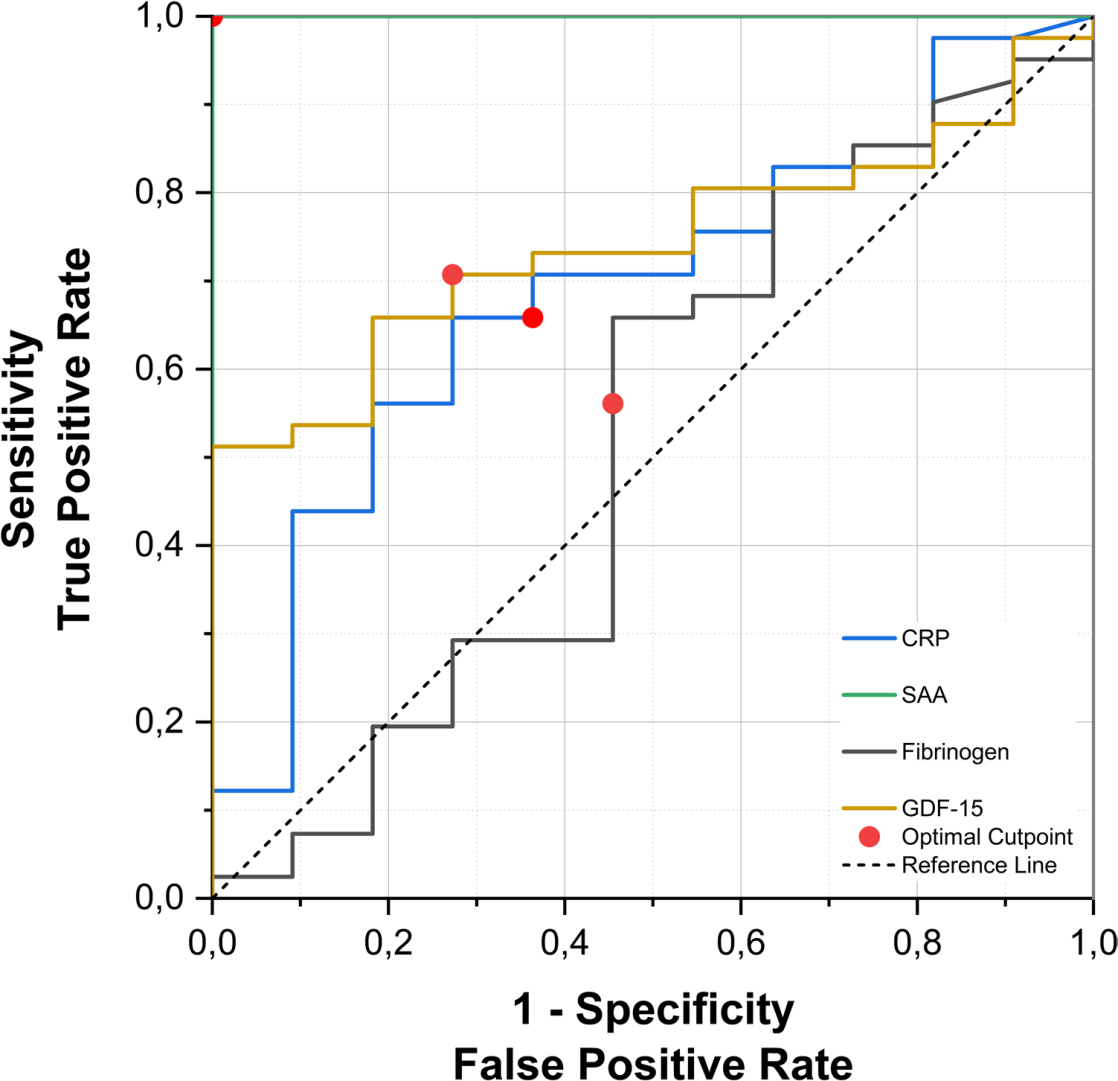
Receiver operating characteristic (ROC) curves of CRP, SAA, fibrinogen, and GDF-15 for identifying subclinical inflammation in FMF patients (*n* = 52). The curves demonstrate the diagnostic performance of each biomarker, with AUC values of 0.691 (95% CI 0.501–0.880; *p* = 0.054) for CRP, 1.000 (95% CI 1.000–1.000; *p* < 0.001) for SAA, 0.522 (95% CI 0.342–0.703; *p* = 0.822) for fibrinogen, and 0.743 (95% CI 0.536–0.950; *p* = 0.014) for GDF-15

ROC analysis was conducted to evaluate the performance of biomarkers in identifying subclinical inflammation among FMF patients. GDF-15 showed a moderate yet statistically significant discriminative ability, whereas CRP demonstrated only limited significance. By contrast, fibrinogen exhibited poor discriminative power and did not reach statistical significance (Fig. 4).

## Discussion

In this study, the finding of significantly elevated serum GDF-15 levels during attack-free periods in FMF patients supports the presence of a clinically silent, biologically active low-grade (subclinical) inflammation. Although GDF-15 correlates with classical inflammatory markers such as CRP and SAA, its elevation in some cases suggests that GDF-15 may provide complementary information on inflammatory burden during the attack-free period, rather than replacing conventional markers. The significant discriminative capacity observed in ROC analysis (AUC: 0.783) suggests that GDF-15 may help identify subclinical inflammatory activity and support monitoring of inflammatory burden during the attack-free period in FMF. Because adiposity can influence inflammatory biomarkers, BMI represents a potential confounder [30]. However, BMI was comparable between the FMF and control groups in our cohort (Table 1), reducing the likelihood of BMI-related confounding; however, residual confounding cannot be entirely excluded.

Regarding cohort representativeness, the proportions of fever and abdominal pain in our patients appear lower than those reported in some FMF cohorts, including pediatric series [31]. This may reflect differences in case mix and data capture, as these frequencies are based on attack-related manifestations recorded in patient histories and medical records rather than on symptoms at the time of blood sampling (Table 2). In addition, enrollment occurred in an outpatient setting during the attack-free period, and all patients received colchicine. Because patients using systemic corticosteroids and/or immunosuppressive biologic therapies were excluded, our cohort may under-represent more severe or colchicine-resistant FMF requiring biologic therapy.

GDF-15 is a pleiotropic cytokine that increases in response to various pathophysiological processes, including mitochondrial stress, tissue injury, cellular strain, and inflammation [32]. At the molecular level, GDF-15 is a stress-responsive cytokine induced by inflammatory signaling and cellular stress—particularly mitochondrial dysfunction and oxidative stress—and has been proposed as a systemic readout of cellular strain [13, 17]. In the literature, elevated GDF-15 levels have been demonstrated in many chronic inflammatory diseases, including cardiovascular diseases, chronic kidney failure, rheumatoid arthritis, and systemic lupus erythematosus [17, 33, 34].

FMF develops as a result of excessive activation of the pyrin inflammasome, driven by mutations in the MEFV gene, and is characterized by increased release of proinflammatory cytokines—particularly IL-1β [35–37]. In this process, innate immune cells such as macrophages and neutrophils play active roles; notably, neutrophil extracellular traps (NETs) are key mechanisms that both sustain inflammation and contribute to tissue damage [38]. In this respect, FMF differs from these other diseases in that it arises from dysfunction of the innate immune system, owing to its autoinflammatory nature [39, 40]. Against this biological background, elevated GDF-15 during attack-free periods may reflect persistent low-grade inflammatory and cellular stress (including mitochondrial/oxidative stress in activated innate immune cells) that may not be fully captured by classical acute-phase reactants, providing a plausible mechanistic basis for its potential association with subclinical inflammatory burden in FMF [13, 17]. Within this distinct pathogenesis, the elevation of GDF-15 suggests that alternative pathways independent of classical inflammatory mediators may also be activated. Although SAA served as the operational criterion for subclinical inflammation in our analyses, GDF-15 may not always parallel conventional acute-phase reactants (including SAA/CRP), suggesting that it may provide complementary information on inflammatory burden during the attack-free period. Clinically, we do not propose GDF-15 as a replacement for established markers such as SAA; rather, it may be considered an adjunct marker to (i) identify discordant clinical-laboratory profiles and (ii) support longitudinal monitoring of inflammatory burden during the attack-free period.

In our cohort, GDF-15 levels were not significantly associated with MEFV mutation subgroups or M694V mutation status. While certain mutations, particularly M694V homozygosity, have been linked to more severe clinical phenotypes [41], our findings suggest that GDF-15 may not be primarily determined by genotype during attack-free periods and may have potential utility in the monitoring of subclinical inflammation.

In the literature, a study employing noninvasive methods demonstrated a significantly increased prevalence of liver fibrosis in FMF patients [42]. It has been reported that conditions which may culminate in hepatic fibrosis—such as nonalcoholic fatty liver disease (NAFLD) and cryptogenic cirrhosis—can occur in FMF, and that recurrent inflammation may play a role in their development [43]. In our study, consistent with the literature, FMF patients exhibited significantly higher levels of CRP, fibrinogen, SAA, uric acid, ALP, ALT, AST, GGT, and the APRI fibrosis score compared with controls. These findings indicate that ongoing systemic inflammation affects hepatic and metabolic profiles.

In our study, increases were observed in hematologic parameters—RDW, WBC, and monocyte count—in FMF patients. These findings suggest that hematologic reflections of the inflammatory process can also be monitored clinically. In the literature, RDW is significantly higher in FMF patients than in controls and is associated with subclinical inflammation as a subgroup finding [3]. Another study reported that RDW is higher in FMF patients, both compared with controls and particularly among those carrying the M694V mutation [44]. Evaluating these parameters alongside potential adjunct biomarkers such as GDF-15 may enable a more accurate assessment of inflammatory burden during attack-free periods in FMF and help improve therapeutic strategies.

## Conclusion

This study showed that GDF-15 levels were significantly elevated in FMF patients and may provide complementary information to classical inflammatory markers in the assessment of subclinical inflammation during attack-free periods. However, to enhance the clinical applicability of these findings, evaluations in larger cohorts encompassing different FMF phenotypes—with longitudinal follow-up and assessment in relation to colchicine response—are required. Prospective, multicenter studies will provide more robust evidence.

## Limitations and recommendations

This study has several limitations. First, although the control group was matched to the FMF cohort by age and sex, menopausal status in female participants was not considered, and the phase of the menstrual cycle at sampling was not recorded. Given that hormonal fluctuations can affect inflammatory and metabolic parameters, this may represent a potential source of bias. Second, while the sample size was adequate to detect medium effect sizes, it limits the generalizability of subgroup analyses, particularly those stratified by MEFV mutation status and subclinical inflammation. Third, the cross-sectional design does not allow assessment of temporal changes in GDF-15 levels or evaluation of their predictive value for long-term outcomes such as amyloidosis and renal involvement. Fourth, the interval since the last FMF attack was not captured in a standardized manner in this cross-sectional dataset; therefore, the median time since the last attack could not be reported. Finally, the single-center nature of the study restricts external validity, underscoring the need for larger, multicenter investigations.

## Data Availability

The original contributions presented in this study are included in the article; further inquiries can be directed to the corresponding author.
